# The influence of spatial frequency content on facial expression processing: An ERP study using rapid serial visual presentation

**DOI:** 10.1038/s41598-018-20467-1

**Published:** 2018-02-05

**Authors:** Jinhua Tian, Jian Wang, Tao Xia, Wenshuang Zhao, Qianru Xu, Weiqi He

**Affiliations:** 1 Research Center of Brain and Cognitive Neuroscience, Liaoning Normal University, Dalian, 116029 China; 2 School of Public Policy and Management, Anhui Jianzhu University, Hefei, 230061 China

## Abstract

Spatial frequency (SF) contents have been shown to play an important role in emotion perception. This study employed event-related potentials (ERPs) to explore the time course of neural dynamics involved in the processing of facial expression conveying specific SF information. Participants completed a dual-target rapid serial visual presentation (RSVP) task, in which SF-filtered happy, fearful, and neutral faces were presented. The face-sensitive N170 component distinguished emotional (happy and fearful) faces from neutral faces in a low spatial frequency (LSF) condition, while only happy faces were distinguished from neutral faces in a high spatial frequency (HSF) condition. The later P3 component differentiated between the three types of emotional faces in both LSF and HSF conditions. Furthermore, LSF information elicited larger P1 amplitudes than did HSF information, while HSF information elicited larger N170 and P3 amplitudes than did LSF information. Taken together, these results suggest that emotion perception is selectively tuned to distinctive SF contents at different temporal processing stages.

## Introduction

Throughout evolution, humans have developed the ability to detect and respond to certain challenges and opportunities^1,2^, especially under the condition of limited attentional resources (i.e., in an emergency situation). The rapid decoding of visual information helps us to identify the affective states of other people and apply appropriate behavioral strategies. Moreover, different channels of spatial frequencies (SFs), which represent various periodic luminance variations across space, have different influences on the processing of visual stimuli^3,4^. Influential models of visual recognition have postulated that SF contents may follow a specific temporal hierarchy in visual object recognition^5–7^. These models suggest that the visual system processes visual input by following a predominantly coarse-to-fine strategy (from LSFs to HSFs), which can facilitate the rapid extraction of visual information.

Additionally, studies on SF have provided some evidence for the mechanism underlying emotional face processing^8–11^. The dual-route model of emotion processing suggested that there are two parallel routes for the processing of emotional information: a subcortical “low road” that provides fast, but crude, biologically significant signals to the amygdala, and a longer, slower “high road” that processes detailed information through cortical visual areas^2,12,13^. In support of this model, Vuilleumier, *et al*.^14^ found larger amygdala and subcortical (pulvinar and superior colliculus) activation for LSF, but not for HSF information in fearful expression perception, suggesting a functional role for the subcortical pathway in providing coarse and threat-related signals. This finding is consistent with neural computational studies which have shown that LSF content, as compared with HSF content, provides more efficient information for the categorization of threat-relevant faces^15,16^.

In contrast, there is an emerging view that the processing of affective visual stimuli relies on both LSF and HSF information, and that the dual-route model needs to be revised to a more flexible model - the multiple-waves model^17^. This model postulates that multiple cortical regions, as well as subcortical structures, play a prominent role in the processing of ecologically relevant signals^17–20^. A study in a patient with a damaged amygdala showed that the patient’s impaired recognition of fearful faces was due to the impaired processing of the eye region of faces conveying HSF information^21^. This finding demonstrates the importance of HSF information in decoding fearful expressions and implies that the amygdala is involved in this type of visual processing. Furthermore, a number of psychophysical studies have suggested that participants primarily use HSF rather than LSF information to discriminate fearful faces from other expressions^10,18,22^.

In contrast to fMRI and behavioral studies, event-related potential (ERP) techniques offer high time resolution, and the temporal characteristics of emotional face processing have been explored in numerous ERP studies. Three of the most prominently studied ERP components are P1, N170, and P3. P1 is an early visual component detected at lateral occipital electrodes, which reflects a fast, exogenous response to visual stimulation^23,24^. The face-sensitive N170 component reflects the encoding of the structure and configuration of faces^25,26^. The late P3 component is sensitive to stimulus valence, reflecting a more elaborate processing of emotional information^27^.

However, the results of ERP studies on SF processing remain inconclusive. A number of ERP studies have suggested that LSF information of threat-related stimuli (faces and scenes) can be extracted rapidly at early stages of emotional processing, as reflected by the ERP components P1 and/or N170^28–31^. In addition, an intracranial event-related potential (iERP) study found a fast amygdala response (starting 74 ms post stimulus onset) especially to LSF fearful faces, providing direct evidence for the existence of the fast, subcortical pathway to the amygdala^32^. However, You and Li^33^’s study on the processing of SF-filtered threat scenes found a significant SF-emotion interaction on P1, suggesting that fear and disgust evoke opposite response patterns in LSF (localized in the dorsal visual stream) and HSF (localized in the ventral visual stream) conditions. This result indicates that both HSF and LSF information can affect the perception of threat.

The rapid serial visual presentation (RSVP) paradigm represents an appropriate task to investigate the time course of emotion processing in the context of limited attentional resources^34–37^. In this paradigm, a series of stimuli are presented sequentially and rapidly (6–20 items per second), and when the interval is around 200–500 ms, the detection of the second target (T2) is impaired by the correct detection of the first target (T1). This phenomenon is called attentional blink (AB)^38,39^, and can be efficiently used to detect whether limited attentional resources affect the response accuracy of different emotional stimuli. Luo, *et al*.^34^ combined the RSVP task with emotional facial expressions (from the Chinese Facial Affective Picture System, CFAPS) and proposed a three-stage model of emotional facial expression processing. In this model, the brain distinguishes threat-relevant facial expressions (fear) from others at the first stage, which explains the augmented early visual ERP component P1/N1, elicited by fearful expressions compared with happy or neutral expressions. At the second stage, the brain distinguishes emotional (happy and fearful) and neutral facial expressions, reflected by N170/VPP. The third stage is differentiating between happy, neutral, and fearful facial expressions, as reflected by P3/N3. Interestingly, subsequent research has shown that the processing of emotional words and scenes also show similar patterns^35–37,40^, but the latter two processing stages are more general and stable^36,37^.

Prior studies related to SF processing have tended to focus on negative emotion processing. There are very few studies have investigated the time course of SF information in the decoding of both positive and negative facial expressions. Therefore, we used RSVP paradigm to further explore the processing of SF-filtered images of happy, fearful, and neutral facial expressions with EEG measurement. Previous studies have suggested that the P1, N170, and P3 components are significantly affected by emotional valence^34,35^, and emotional face processing is mediated by specific SF contents^9,31^. Taking these presumptions into consideration, we hypothesized that the early perception of emotional facial expressions relies on specific SF information, and we expected to observe different neural responses to happy, neutral, and fearful faces in early ERP components (P1 and/or N170). Furthermore, we also hypothesized that different SF contents would elicit different neural processing patterns, and we expected to observe different responses to LSF and HSF information in each of the P1, N170, and P3 components.

## Results

### Behavioral performance

ANOVAs for the response accuracy revealed significant main effects of facial expression and SF (*F*_2,58_ = 12.902, *p* < 0.001, η^2^p = 0.308; *F*_1,29_ = 26.446, *p* < 0.001, η^2^p = 0.477). Participants performed better in the HSF condition (95.8 ± 0.6%) than in the LSF condition (92.0 ± 0.8%, *p* < 0.001). Pairwise comparison of the main effect of facial expression showed that the accuracies of fearful (96.0 ± 0.6%, *p* < 0.001) and happy faces (95.1 ± 0.8%, *p* = 0.008) were higher than that of neutral faces (90.5 ± 1.2%), while the former two emotion conditions show no significant difference (*p* = 0.750). Furthermore, there was no significant interaction effect between facial expression and SF (*F*_2,58_ = 0.900, *p* = 0.386, η^2^p = 0.030).

### ERP data analysis

#### P1

**Figure 1:**
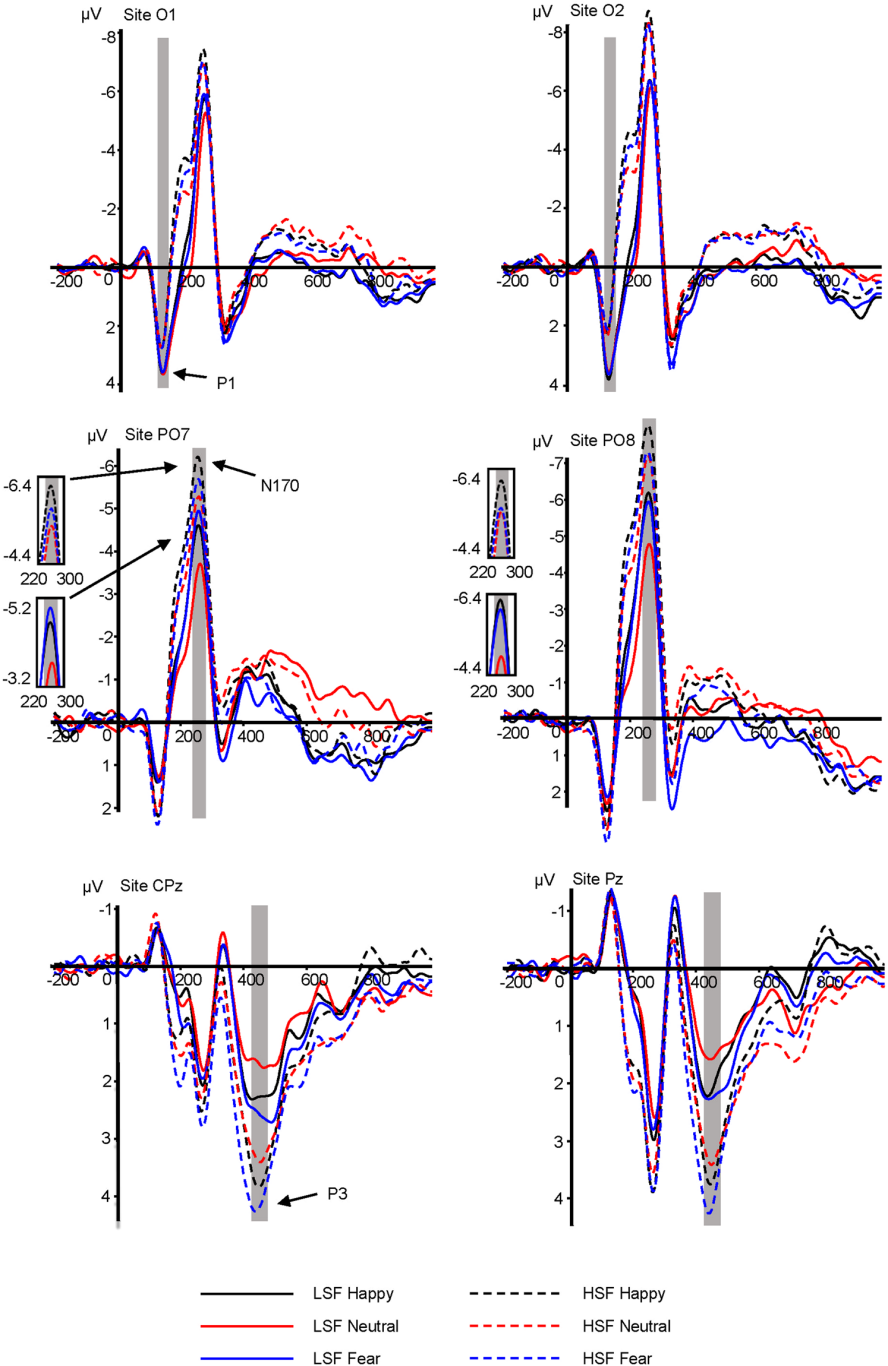
Grand average ERPs for LSF happy, LSF neutral, LSF fearful, HSF happy, HSF neutral, and HSF fearful faces recorded at the indicated electrode sites. The gray area represents the time window of each component.

The P1 amplitude showed significant main effect at SF and electrode (*F*_1,29_ = 8.914, *p* = 0.006, η^2^p = 0.235; *F*_5,145_ = 12.645, *p* < 0.001, η^2^p = 0.304; Fig. 1); the main effect of facial expression was not significant (*F*_2,58_ = 1.027, *p* = 0.357, η^2^p = 0.034). LSF faces (3.072 ± 0.253 μV) elicited larger P1 amplitudes than did HSF faces (2.015 ± 0.392 μV, *p* = 0.006). Largest amplitudes were elicited at O1 (3.223 ± 0.312 μV). Bilateral occipital electrode sites O1 and O2 (2.750 ± 0.274 μV) elicited larger P1 amplitudes than did central occipital electrode sites POz (1.636 ± 0.333 μV, *p* < 0.01) and Oz (2.049 ± 0.312 μV, *p* < 0.01).

#### N170

**Figure 2:**
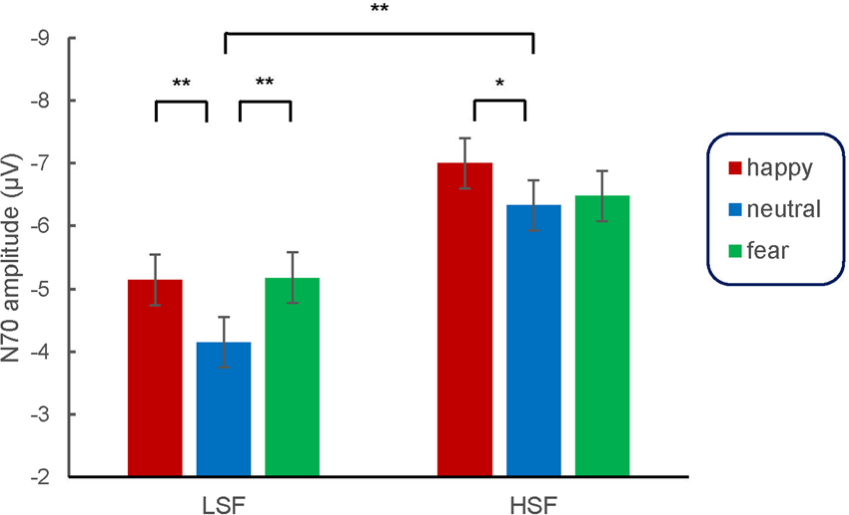
The interaction effect of facial expression by SF on the N170 amplitude. Bars represent the standard error of the mean. *p < 0.05; **p < 0.01.

Facial expression, SF, and electrode each had a significant effect on the N170 amplitude (F_2,58_ = 11.214, *p* < 0.001, η^2^p = 0.279; *F*_1,29_ = 32.654, *p* < 0.001, η^2^p = 0.530; *F*_3,87_ = 7.826, *p* = 0.003, η^2^p = 0.213). Happy (−6.071 ± 0.417 μV, *p* < 0.001) and fearful faces (−5.827 ± 0.432 μV, *p* = 0.011) elicited larger amplitudes than did neutral faces (−5.240 ± 0.415 μV), and HSF content (−6.601 ± 0.477 μV) elicited larger amplitudes than did LSF content (−4.824 ± 0.393 μV, *p* < 0.001). Larger amplitudes were elicited at the PO7 (−5.549 ± 0.541 μV, *p* = 0.001) electrode sites and the PO8 (−6.954 ± 0.570 μV, *p* = 0.002) electrode than at the P7 (−4.318 ± 0.415 μV) sites. The SF × Emotion interaction was also significant (*F*_2,58_ = 4.527, *p* = 0.017, η^2^p = 0.135). The further simple effects analyses revealed that happy (−5.144 ± 0.391 μV, *p* = 0.001) and fearful faces (−5.177 ± 0.455 μV, *p* = 0.001) elicited larger amplitudes than did neutral faces (−4.152 ± 0.407 μV) in the LSF condition; happy faces (−6.998 ± 0.507 μV, *p* = 0.010) elicited larger amplitudes than did neutral faces (−6.329 ± 0.504 μV), while the difference between fearful (−6.476 ± 0.465 μV) and neutral faces was not significant (*p* = 1.000) in the HSF condition (Figs 1 and 2).

#### P3

**Figure 3:**
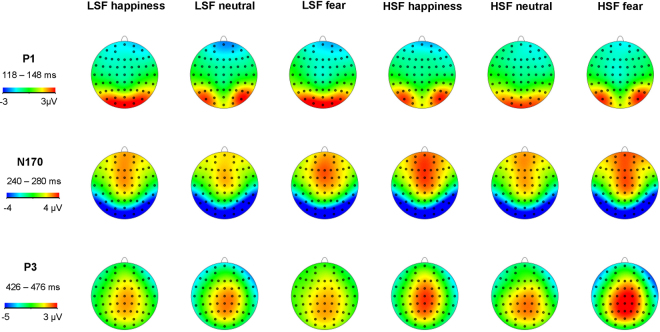
Grand average ERP topographies of the P1, N170, and P3 components across two SFs and three emotion conditions.

The P3 amplitude showed significant main effect at facial expression, SF and electrode (*F*_2,58_ = 25.275, *p* < 0.001, η^2^p = 0.466; *F*_1,29_ = 23.049, *p* < 0.001, η^2^p = 0.443; *F*_5,145_ = 7.623, *p* < 0.001, η^2^p = 0.208). There was no significant interaction effect between facial expression and SF (*F*_2,58_ = 0.582, *p* = 0.552, η^2^p = 0.020). CPz (2.763 ± 0.351 μV) elicited largest amplitudes of P3, and centro-parietal electrode sites CPz and Pz (2.520 ± 0.359 μV) elicited larger P3 amplitudes than did bilateral electrode sites CP3 (1.501 ± 0.269 μV) and CP4 (1.776 ± 0.232 μV) (*p* < 0.05). HSF faces (2.553 ± 0.281 μV) elicited larger P3 amplitudes than did LSF faces (1.587 ± 0.246 μV, *p* < 0.001). The pairwise comparison showed that happy (2.122 ± 0.288 μV, *p* < 0.001) and fearful (2.460 ± 0.313 μV, *p* < 0.001) faces elicited larger P3 amplitudes than did neutral faces (1.486 ± 0.223 μV), and fearful faces elicited lager amplitudes than did happy faces (*p* = 0.012). The scalp topographies of the grand average ERPs across two SFs and three emotional conditions are shown in Fig. 3.

## Discussion

In this study, we used ERPs and an RSVP task with SF-filtered happy, fearful, and neutral faces, to explore the time course of neural dynamics involved in the processing of facial expression conveying specific SF information.

The behavioral results show an advantage for the processing of emotional expressions (happy and fearful faces), which is in line with the common emotion effect evident in the prioritized processing of negative and positive stimuli during a deficit of attentional resources, presumably due to their biological significance^41–43^. Furthermore, response accuracy on HSF stimuli was better than on LSF stimuli. Compared to previous research on sad and happy face categorization^9,44^, our results show no SF-emotion interaction in response accuracy. This difference may be due to the different presentation times of visual stimuli in different tasks, which can affect the accuracy of coarse/fine object recognition^45–47^. More precisely, LSF information is more effective for the object recognition in a short presentation time and HSF information requires a longer exposure duration to influence participant performance. Considering that the presentation time of the target stimuli was invariant in the present RSVP task, our discussion therefore focuses on the neural time course of face processing rather than the behavioral results.

The ERP results show the time course of LSF and HSF information in the decoding of facial expression. The early visual-sensitive P1 component showed that LSF faces yielded larger neural response than did HSF faces. Considering that we carefully equalized the low-level characteristics of the images across emotion conditions, this result reveals that P1 is sensitive to SF information processing^31,48^. Unlike earlier research^34,40^, we did not find a negative bias in P1 for fearful faces, which indicated that fearful facial expressions cannot be distinguished from other expressions at the early stage in both LSF and HSF conditions. One possible explanation is that the SF filter impaired the configural and feature information of the face stimuli^9,49,50^, and that the SF-filtered images are less salient and emotionally aroused than broadband images.

The face-sensitive N170 component showed that both fearful and happy faces elicited larger amplitudes than did neutral faces in the LSF condition, while happy but not fearful faces elicited larger amplitudes than did neutral faces in the HSF condition. In other words, the brain can distinguish happy and fearful from neutral facial information in the LSF condition, but can only distinguish happy from neutral information in the HSF condition. The LSF N170 result pattern is consistent with the second stage of Luo’s model, while the HSF results are not. These results indicate that LSFs are important for the rapid detection of facial expressions, while HSFs do not represent efficient information for the fast detection of fear^31^. Moreover, both fearful and happy face processing are mediated by LSF content at the early stage of emotion processing, which is in accordance with the idea that coarse SF contents of visual stimuli convey biologically significant information^32^. On the other hand, the rapid decoding of happy faces was also shown in HSF information processing, which argue against the common assumption that fast emotional response can be elicited only when LSF information of an emotional stimulus is presented^14^. What is more, we find that happy face categorization was less impacted in the absence of certain SF channels, which could explain why happy faces are recognizable over a variety of different viewing distances, when there is either LSF or HSF information available^18,51^.

The P3 component differentiated between the three types of emotional faces in both LSF and HSF conditions, which is in line with the third stage of Luo’ model. This finding indicates that the brain distinguishes emotion information as positive or negative at later stages, irrespective of SF information. In other words, both happy and fearful emotion information are extracted from LSF and HSF contents at this stage.

Taken together, the divergent N170 effect and the similar P3 effect clearly indicate that the discrimination of different emotional facial expressions relies on both LSF and HSF information, but that these parallel or converging pathways process information at different speeds. These results provide evidence for the existence of parallel visual pathways in emotion processing, which is consistent with a recent proposal of multi-path processing of emotion^17^.

Moreover, ERP components are also mediated by SF contents. At the early stage of emotional processing, as reflected by P1, LSF content elicited larger amplitudes than did HSF content. However, the results were reversed at later emotion processing stages: HSFs elicited larger N170 and P3 amplitudes than did LSFs. This SF effect in facial expression is consistent with the sequential processing (from coarse to fine) of SF information in visual object recognition^5–7^.

## Conclusion

Through the use of an RSVP paradigm and the ERP technique, we find that the processing and detection of emotional information in a face is mediated by SF contents. LSF information elicits larger amplitudes in the early P1 component than does HSF information, while HSF information elicits larger amplitudes in the later N170 and P3 components than does LSF information. Furthermore, the face-sensitive N170 component is modulated by both facial expressions and SF channels, while P3 shows similar emotion processing patterns irrespective of SF information. More specially, the N170 effect indicates that the rapid encoding of fearful facial expression is primarily mediated by LSF information, while the fast detection of happy facial expression involves both LSF and HSF information.

## Methods

### Participants

Thirty healthy undergraduate students (15 men and 15 women; mean age 19.6 years, SD = 1.59 years) from Chongqing University of Arts and Science were volunteered to participate in the experiment. All participants were healthy, right-handed, without psychiatric disorders history, and had normal or corrected normal vision. This study was approved by the Ethics Committee of Chongqing University of Arts and Sciences, and written informed consent was obtained from all participants before the experiment. All methods mentioned in this research were performed in accordance with the relevant guidelines and regulations.

### Stimuli

Materials consisted of 48 face pictures (36 SF-filtered upright faces and 12 inverted faces) and 3 upright house stimuli. The original broadband face pictures were selected from the native Chinese Facial Affective Picture System (CFAPS), including 6 happy faces, 6 fearful faces, 6 neutral faces, and 12 inverted neutral faces. Please see the supplementary materials for the reason why we excluded the broadband images in this study. Furthermore, the reason why we chose only 36 target face stimuli for this study and the analysis of the training effect are also provided in the supplementary materials.

Valence and arousal ratings (ranging from 1: extremely unpleasant/extremely arousing to 9: extremely pleasant/not at all arousing) were provided by an independent sample (*N* = 24). The one-way repeated measures ANOVA of valence and arousal showed that the face pictures differed significantly (*F*_2,46_ = 204.595, *p* < 0.001, η^2^p = 0.899; *F*_2,46_ = 132.540, *p* < 0.001, η^2^p = 0.886). Valence ratings confirmed that fearful faces (3.208 ± 0.142) indeed elicited more unpleasant than did neutral (4.972 ± 0.049, *p* < 001) and happy faces (7.097 ± 0.171, *p* < 0.001), and happy faces elicited more pleasant than did neutral faces (*p* < 001). Arousal ratings indicated that the highest arousal was induced by fearful facial expression (6.403 ± 0.235), followed by happy faces (6.306 ± 0.265), and the least by neutral faces (2.285 ± 0.233) (the former two showed no significant difference, *p* = 1.000).

To generate the LSF and HSF face images, the original photograph were transformed to grayscale and equal size (260 × 300 pixels), normalized to equal luminance, and low-pass filtered at 2 cycles per degree or high-pass filtered at 6 cycles per degree, respectively. These cutoff parameters were based on previous literature^31,52–56^. Filtering was performed in Matlab (Mathworks, Natick, MA), using a set of two order Butterworth filter. After the filtering, we used SHINE toolbox^57^ to ensure all the filtered image sets were equaled on luminance [92 (0.07) on a 0–255 scale] and contrast [64(0.13)]. All stimuli were presented on a 17-inch LCD monitor (resolution: 1280 × 800; refresh rate: 60 Hz), 80 cm from the participants’ eyes, and with the viewing angle as 7.6 × 9°.

### Procedure

**Figure 4:**
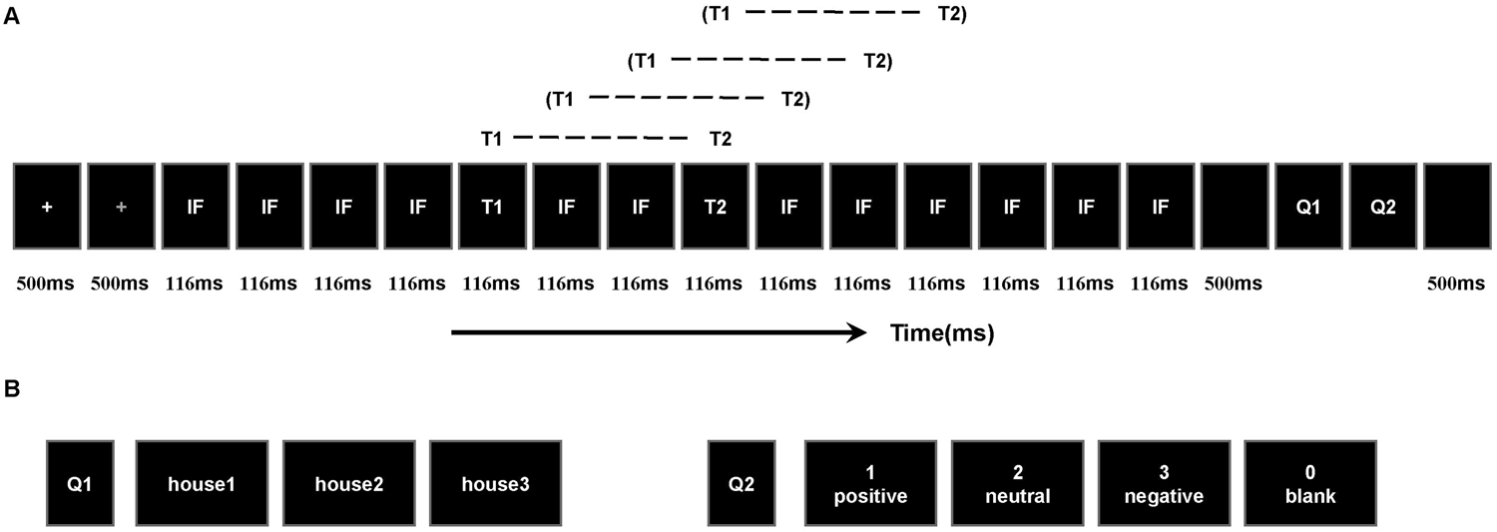
Examples of the stimuli and the RSVP paradigm used in this experiment. (**A**) Each trial contained 12 inverted faces (IF) and two target stimuli (T1 and T2), followed by two questions (Q1 and Q2) at the end of each trial. The T1 emerged randomly and equiprobably at the fifth, sixth, seventh position, T1 and T2 were presented within an interval of 232 ms. (**B**) Example for the two questions in each trial.

To investigate the time course of LSF or HSF information in decoding facial expression, we chose a dual-target RSVP paradigm^34–37^ for this study. Participants were seated in a dimly lit, quiet room and were presented with four experimental blocks of 120 trials each. As shown in Fig. 4, each trial began with a white crosshair and a blue crosshair at the center of the screen, each presented for 500 ms, following by 14 pictures (including two target stimuli) with a stimulus-onset asynchrony (SOA) of 116 ms and no blank inter-stimulus interval (ISI). The first target stimulus (T1) emerged randomly and equiprobably at the fifth, sixth, or seventh position, nextly, two distracting stimuli appeared, then the second target stimulus (T2, SOA = 232 ms), and other distracting stimuli (inverted face pictures) items were presented. All stimuli were presented in the center of the screen. To obtain the ERP components purely elicited by T2, we designed a baseline condition (a blank black screen) to remove the superposed electrical activity elicited by the distractors. The T1 (task: recognize the house that was memorized before the experiment, press key “1”, “2”, “3”, correspondingly) was one of the three house pictures and the T2 (task: discriminate the valence in the picture, press key “1” if T2 was positive, key “2” when neutral, key “3” when negative, key “4” if T2 was absent) was one of the 36 filtered face pictures. The question would not disappear until participants pressed the index key or until 3000 ms elapsed. All subjects were required to respond to the two questions with their right hand. Stimulus presentation was controlled by E-Prime 2.0 software (Psychology Software Tools Inc., Pittsburgh, PA, USA).

### Electrophysiological recording and analysis

During task performance, the Electroencephalogram (EEG) (sampling rate of 500 Hz) was recorded from a 64-channel amplifier using a standard 10–20 system (Brain Products, Gilching, Germany), with the reference on right and left mastoids. The vertical electrooculogram (EOG) was recorded from electrodes placed blow the left eye. All electrode impedances (EEG and EOG) were maintained below 5 kΩ.

The EEG data were analyzed offline for ERPs using Brain Vision Analyzer software (Brain Products, Munich, Germany). We employed an off-line re-reference to the averaged reference and filtered the data with a 30 Hz, 24 dB/octave low pass filter. ERP epochs were extracted beginning 200 ms before and ending 1000 ms after T2 stimulus, and trials were accepted only if the answer of both T1 and T2 were correct. The 200 ms pre-stimulus was used as baseline to each epoch. Thereafter, trials with EEG voltages exceeding ±75 μV (relative to baseline) were excluded from analysis. Averaged ERPs were computed for six stimulus conditions [Emotion (happy, neutral, fear) × SF (LSF, HSF)]. According to the topographical distribution and previous research^34,35^, the P1, N170, and P3 components were chosen for statistical analysis in the present study. Based on the approach of collapsed localizer^58,59^, the data were collapsed across all the conditions to extract the time windows and electrode sites for each component. Time windows for amplitude calculation were centered at the peak latencies of the collapsed waveforms, with a shorter window length for earlier ERP components and a longer length for later components. More specifically, the mean amplitude of P1 component (time window: 118–148 ms) was measured and analyzed at the following six electrode sites (O1, O2, PO3, PO4, POz, and Oz); P7, P8, PO7, and PO8 were selected for statistical analysis of the N170 component (time window: 240–280 ms); P3 (time window: 426–476 ms) were analyzed at CPz, Pz, P3, P4, CP3, and CP4 electrode sites. Mean amplitudes of each component were subject to a three-way repeated measures analyses of variance (ANOVAs; with Greenhouse-Geisser corrections) with SF (two levels: HSF, LSF), emotion (three levels: happy, neutral, fear), and electrode position. Significant ANOVA effects were followed by pairwise comparisons to contrast the effects of individual conditions (using the Bonferroni method).

## Electronic supplementary material

Supplementary Information
