# Supplementary material for: The influence of spatial frequency content on facial expression processing: An ERP study using rapid serial visual presentation
Source: Sci Rep. 2018 Feb 5;8:2383. doi: 10.1038/s41598-018-20467-1 (PMC5799249; doi:10.1038/s41598-018-20467-1)
Supplement: Supplementary file 1 — Supplementary Information [file 41598_2018_20467_MOESM1_ESM.pdf]

The influence of spatial frequency content on facial expression processing: An ERP study using rapid serial visual presentation

Jinhua Tian[1#], Jian Wang[2#], Tao Xia[1], Wenshuang Zhao[1], Qianru Xu[1], Weiqi He[1*]

[1] Research Center of Brain and Cognitive Neuroscience, Liaoning Normal University, Dalian 116029, China;

[2] School of Public Policy and Management, Anhui Jianzhu University, Hefei 230061, China;

#Co-first authors

*Corresponding author

Correspondence and requests for materials should be addressed to W.H. (E-mail: weiqi79920686@sina.com)

Supplementary Materials

More information about the stimuli

The present study specifically aimed at investigating the functional role of LSF and HSF information in emotional face processing. As with previous studies [1,2], this study did not include broadband images. To our knowledge, the joint contribution of LSF/HSF information on facial emotion recognition has been widely investigated and it should better be addressed by measuring behavioral and neural responses to visual information while SF content of an image is parametrically shifted from low to high SFs [3,4]. Moreover, RSVP is a heavy task requiring a large amount of attention resources and too many trials would make the participant tired. Taking all these into account, we have excluded the unfiltered faces in this study.

Target face stimuli consist of 36 face pictures. The choice of face stimuli is based on previous RSVP studies [5,6], and we used only six faces per condition. It is because the Chinese Facial Affective Picture System (CFAPS) contains a total of 880 faces but only 60 fearful faces. To investigate the pure effect of emotional valence (happy, neutral, and fear), we chose the faces with significantly different valence and similar arousal ratings. Only a few faces met these criteria.

The training effect

To examine whether the training effect can influence participant performance, we divided the data into the first half of the trials and the second half of the trials. The

difference between these two half trials was served as the effect of "training" and we have analyzed the behavioral and ERP data again.

Behavioral results. A three-way repeated measures analyses of variance (ANOVAs; with Greenhouse-Geisser corrections) with training (the first half, the second half), emotion (fear, happy, and neutral) and SF (LSF, HSF) were performed on response accuracy. The results revealed significant main effects of training, facial expression and SF ($F_{1,29}$ = 12.764, p = .001, η^2p = .306; $F_{2,58}$ = 12.616, p < .001, η^2p = .303; $F_{1,29}$ = 22.085, p < .001, η^2p = .432). Participants performed better in the latter half trials (90.9 ± 0.6%) than in the former half trials (87.5 ± 1.1%, p = .001). Moreover, the training × emotion interaction, the training × SF interaction, and the training × emotion × SF interaction (all p > .30 with effect sizes < 0.1) were not significant.

ERP analysis. Mean amplitudes (averaged cross selected electrodes) of P1, N170, and P3 were subject to a three-way repeated measures ANOVAs with training, emotion, and SF.

P1. The P1 amplitude showed significant main effect at training and SF ($F_{1,29}$ = 13.728, p = .001, η^2p = .321; $F_{1,29}$ = 12.645, p < .001, η^2p = .304). The first half trials (2.893 ± 0.296 μV) elicited larger P1 amplitudes than did the second half trials (2.320 ± 0.298 μV, p = .006). Furthermore, the training × emotion interaction, the training × SF interaction and the training × emotion × SF interaction (all p > .20 with effect sizes <

0.05) were not significant.

N170. Training, emotion, and SF each had a significant effect on the N170 amplitude ($F_{1,29}$ = 20.467, p < .001, η^2p = .414; $F_{2,58}$ = 9.487, p < .001, η^2p = .247; $F_{1,29}$ = 44.144, p < .001, η^2p = .604). The second half trials (-6.270 ± 0.360 μV) elicited larger N170 amplitudes than did the first half trials (-5.415 ± 0.390 μV, p < .001). Moreover, the emotion × SF interaction ($F_{2,58}$ = 5.633, p = .008, η^2p = .163) was significant, while the training × emotion interaction, the training × SF interaction, and the training × emotion × SF interaction (all p > .05 with effect sizes < 0.1) were not significant.

P3. The P3 amplitude showed significant main effect at training, emotion, and SF ($F_{1,29}$ = 9.301, p = .005, η^2p = .243; $F_{2,58}$ = 8.760, p = .001, η^2p = .232; $F_{1,29}$ = 29.940, p < .001, η^2p = .508). The first half trials (3.075 ± 0.314 μV) elicited larger P3 amplitudes than did the second half trials (2.525 ± 0.385 μV, p = .005). Further, the training × emotion interaction, the training × SF interaction, and the training × emotion × SF interaction (all p > .20 with effect sizes < 0.05) were not significant.

The main effects of behavioral and ERP results were not affected by the factor of "training" and none of the interactions involving training were significant (all p > .05 with effect sizes < 0.1), indicating that the repetition of picture stimuli was not affect participant performance.

Reference

1 Vlamings, P. H., Goffaux, V. & Kemner, C. Is the early modulation of brain activity by fearful facial expressions primarily mediated by coarse low spatial frequency information? *J Vis* **9**, 12 11-13, doi:10.1167/9.5.12 (2009).

2 You, Y. & Li, W. Parallel processing of general and specific threat during early stages of perception. *Social cognitive and affective neuroscience* **11**, 395-404, doi:10.1093/scan/nsv123 (2016).

3 Tanskanen, T., Nasanen, R., Montez, T., Paallysaho, J. & Hari, R. Face recognition and cortical responses show similar sensitivity to noise spatial frequency. *Cereb Cortex* **15**, 526-534, doi:10.1093/cercor/bhh152 (2005).

4 Kauffmann, L., Ramanoel, S. & Peyrin, C. The neural bases of spatial frequency processing during scene perception. *Front Integr Neurosci* **8**, 37, doi:10.3389/fnint.2014.00037 (2014).

5 Luo, W., Feng, W., He, W., Wang, N. Y. & Luo, Y. J. Three stages of facial expression processing: ERP study with rapid serial visual presentation. *NeuroImage* **49**, 1857-1867, doi:10.1016/j.neuroimage.2009.09.018 (2010).

6 Zhang, D. *et al.* Three stages of emotional word processing: an ERP study with rapid serial visual presentation. *Social cognitive and affective neuroscience* **9**, 1897-1903, doi:10.1093/scan/nst188 (2014).
